# Dynamic SWIR imaging near the 1950-nm water absorption band for caries lesion diagnosis

**DOI:** 10.1117/1.JBO.26.5.056006

**Published:** 2021-05-25

**Authors:** John T. Tressel, Marwa Abdelaziz, Daniel Fried

**Affiliations:** University of California, San Francisco, California, United States

**Keywords:** dental caries, short wavelength infrared imaging, lesion activity

## Abstract

**Significance:** It is not sufficient to detect caries lesions on tooth surfaces; it is also necessary to measure the activity of the lesions to determine if intervention is needed. Changes in the reflectivity of lesion areas during dehydration with forced air at short wavelength infrared (SWIR) wavelengths can be used to assess lesion activity since these changes represent the evaporation dynamics of water from the lesion.

**Aim:** The aim of this study is to develop new optical methods for assessing lesion activity on tooth surfaces utilizing the strong water absorption band near 1950-nm.

**Approach:** The time-resolved reflectivity of 20 active and arrested caries lesions on the surfaces of human extracted teeth was monitored at 1300 to 2000 nm using broadband light sources and an extended range InGaAs camera during drying with air.

**Results:** Multiple parameters representing the rate of change of the lesion reflectivity correlated with the presence of a highly mineralized outer surface zone indicative of lesion arrest measured with x-ray microtomography (microCT). Performance at 1950-nm was higher than for other wavelengths.

**Conclusions:** This study demonstrates that SWIR imaging near 1950-nm has great potential for the assessment of lesion activity.

## 1 Introduction

Caries lesions can be arrested by the preferential deposition of mineral at the lesion surface, which inhibits diffusion of fluids into the porous lesion.^1^^–^^3^ Since arrested lesions do not need further intervention, the assessment of lesion activity is essential for clinical diagnosis. Many lesions have been arrested or do not require intervention. Even so, it is difficult to identify active lesions with current diagnostic methods. Accurate assessment of lesion activity, depth, and severity is important for determining whether intervention is necessary. Gold standards for lesion assessment such as transverse microradiography and polarized light microscopy either require destruction of the tooth or in the case of microCT are unsuitable for use *in-vivo*. New non-destructive diagnostic tools that can assess lesion activity in a single visit are needed. Effective employment of new optical diagnostic technologies that can exploit the changes in the light scattering of the lesion has great potential for diagnosing the present state of lesions. Therefore, the development of new methods, such as short wavelength infrared (SWIR) imaging, is needed for the clinical assessment of lesion activity to avoid unnecessary cavity preparations.

When lesions become arrested by mineral deposition, or remineralization, in the outer layers of the lesion, the diffusion of fluids into the lesion is inhibited. Hence, the rate of water diffusion out of the lesion or the evaporation dynamics reflects the degree of lesion activity. Since arrested lesions are less permeable to water due to the highly mineralized surface layer, changes in the rate of water loss can be related to changes in lesion structure and porosity. Changes in fluorescence loss,^4^^–^^6^ thermal emission, and SWIR reflectance^7^^–^^13^ during lesion dehydration have been investigated as methods for assessing lesion activity. Normal enamel is transparent at SWIR wavelengths, whereas early demineralization causes increased SWIR reflectance due to scattering.^14^ Water in the pores at the surface of the lesion absorbs the incident SWIR light, particularly at wavelengths greater than 1450 nm, reducing surface scattering and lesion contrast.^15^^,^^16^ Loss of that water due to evaporation produces a marked increase in reflectivity and lesion contrast. *In-vivo* studies have been published utilizing the fluorescence loss of white spot lesions on coronal surfaces^6^ and thermal imaging to assess root caries during dehydration.^17^ There is a negative association between the surface zone thickness and lesion permeability; a small increase in the surface layer thickness of <20  μm can lead to a marked decrease in permeability.^18^ In addition, in a closely related study, the surface zone was removed from arrested lesions, producing a corresponding rise in the permeability, which provided further confirmation of the role of the surface zone in arresting lesions.^19^

Recent studies at wavelengths beyond 1700-nm show that the contrast of demineralization on tooth surfaces continues to increase with increasing wavelength due to decreasing scattering in sound enamel and increasing water absorption.^20^^–^^22^ Contrast is particularly high near the strong water absorption band at 1950 nm. The high contrast of demineralization and the high sensitivity to water absorption suggests that 1950 nm should be ideal for SWIR reflectance dehydration measurements and recent studies of simulated lesions on bovine enamel blocks support this hypothesis.^21^ That study also found that the change in contrast after drying (ΔI) was significantly higher at the 1950-nm broadband source than at other wavelengths in the 1500 to 2340-nm wavelength range.^21^

The purpose of this study is to investigate the use of SWIR reflectance dehydration measurements at 1950 nm to assess the activity of naturally formed caries lesions on extracted teeth.

## 2 Materials and Methods

### 2.1 Tooth Samples and MicroCT

Twenty extracted teeth were selected with interproximal lesions for participation in this study. Teeth were collected from patients in the San Francisco Bay area with approval from the UCSF Committee on Human Research. The teeth were sterilized using gamma radiation and stored in 0.1% thymol solution to maintain tissue hydration and prevent bacterial growth.

**Fig. 1:**
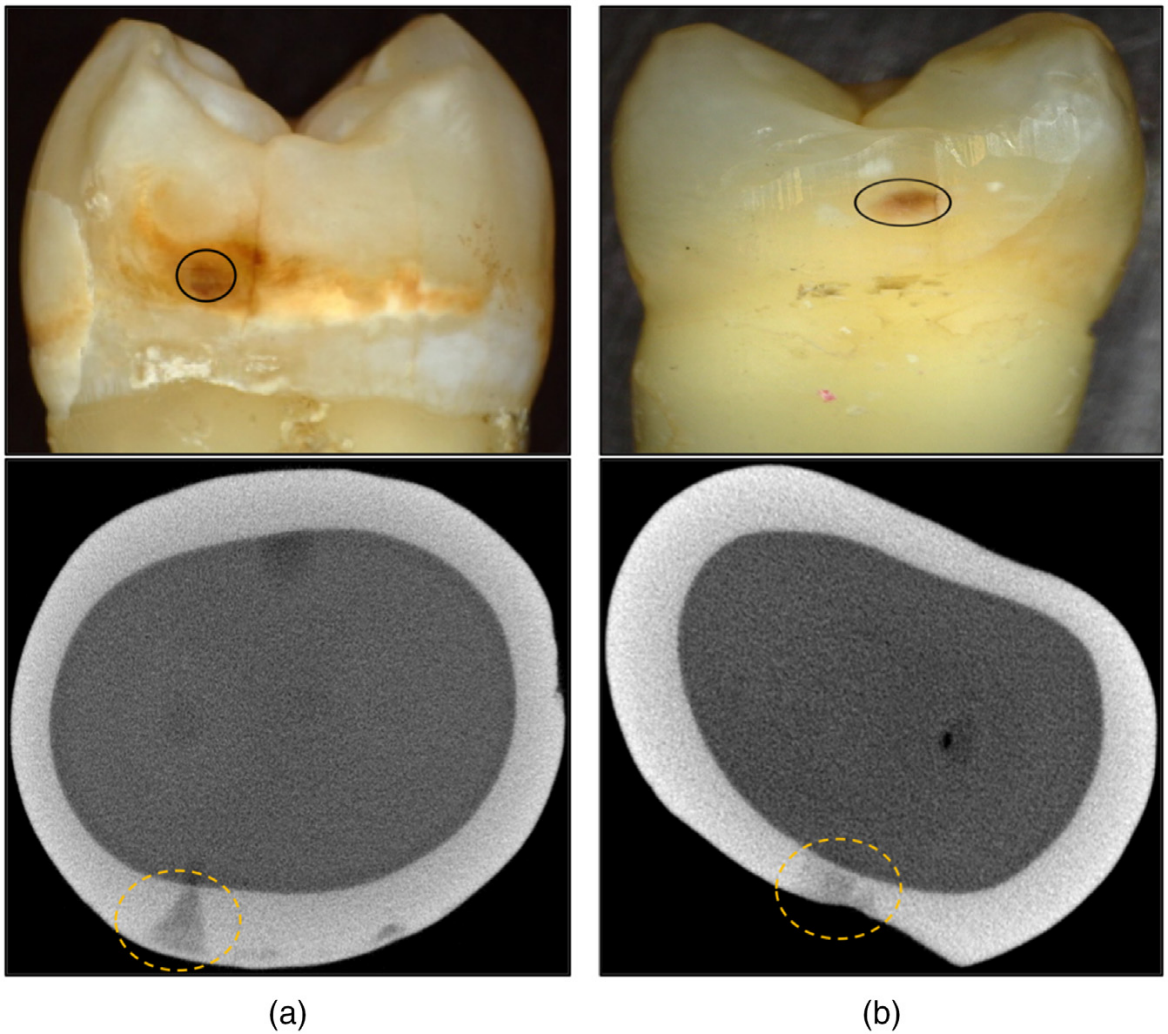
Color (visible) and microCT images of two extracted teeth with suspected (a) arrested and (b) active interproximal lesions. The lesions are enclosed by the solid and dashed circles.

The teeth were imaged using microCT with a 10-μm resolution. A Scanco microCT 50 from Scanco USA (Wayne, PA) located at the UCSF Bone Imaging Core Facility was used to acquire the images. MicroCT images of 120 teeth were examined; ten teeth with interproximal lesions with distinct surface zones of higher mineral content were selected and designated as arrested, while ten teeth for which no discernable surface zones were visible were chosen and designated as active lesions. Figure 1 shows visible and microCT images of an active lesion and an arrested lesion used in the study. The microCT images were extracted orthogonal to the long axis of the tooth, showing the structure of the interproximal lesions and the highly mineralized surface zone that is clearly visible for the arrested lesion. The lesions appear to have similar color, staining, and texture in the visible images; however, the arrested lesion is both larger and deeper than the active lesion. Ten active lesion areas and ten arrested lesion areas were selected on twenty teeth with one lesion area selected per tooth. Often lesions were present on a tooth that was only partially arrested and had both active and arrested areas.^12^

### 2.2 Visible/Color Images

A USB microscope, Model 5MP Edge AM7915MZT, AnMO Electronics Corp. (New Taipei City, Taiwan) equipped with a visible polarizer was used to acquire visible images of all samples. The digital microscope captured 5 mega-pixel (2952×1944) color images. Eight white LED lights contained in the camera illuminated the sample, and a single polarization element was utilized to reduce glare.

### 2.3 SWIR Dehydration Analysis

**Fig. 2:**
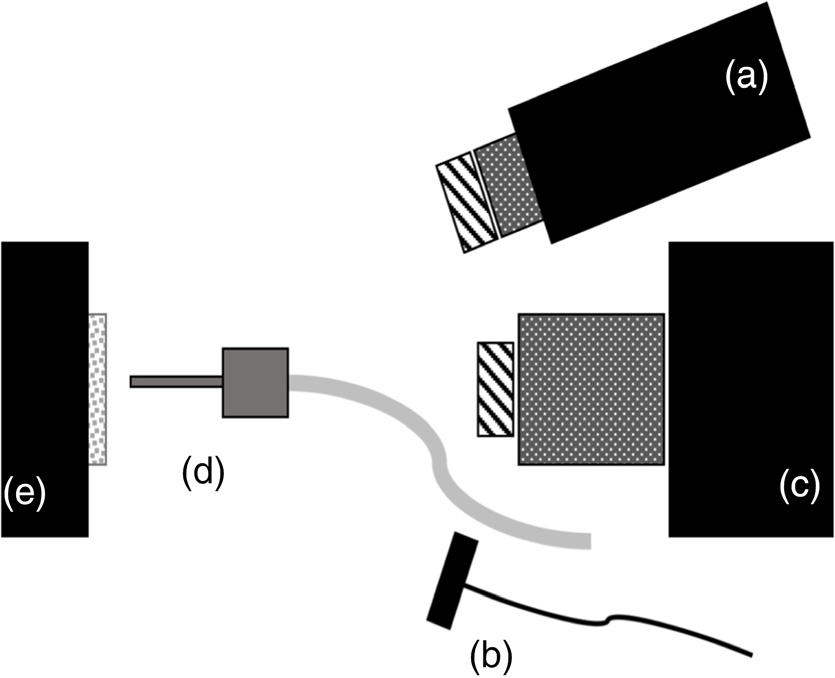
Schematic of the experimental setup showing (a) tungsten-halogen light source with bandpass filters, collimating lens and polarizer; (b) polarized 1950-nm fiber optic light source; (c) Xenics extended range InGaAs camera with lens and polarizer; (d) air nozzle; and (e) tooth samples mounted on XYZ stage. Light sources A and B were positioned on the same side for these measurements.

Samples were stored in a moist environment to preserve internal hydration and were immersed in a water bath before mounting and performing measurements. A computer-controlled air nozzle with a 1-mm aperture and an air pressure set to 25 psi was positioned 4 cm away at a 20-deg angle above the sample plane as shown in Fig. 2.

After each sample was removed from the water bath, an image was captured as an initial reference image, and the pressurized air nozzle was activated to dehydrate the sample. Each measurement consisted of capturing a sequence of images at 25 frames per second for 60 s. For each measurement, the air nozzle and the light source were centered on the region of interest that encompassed the entire sample. The dehydration setup was completely automated using LabVIEW software from National Instruments (Austin, Texas).

A Xenics (Leuven, Belgium) Model Xeva-2.35-320 extended range InGaAs camera sensitive from 900 to 2350 nm (320×240  pixel) was used to acquire the SWIR images. The camera was equipped with a Navitar (Rochester, New York) f=35-mm SWIR optimized (f/1.4) lens, and a 60-mm achromat lens was positioned 40 mm from the 35-mm lens. A high extinction polarizer was used to acquire cross-polarization images from 1500 to 2060 nm. The quantum efficiency peaks at 1500 nm near 65%, drops off rapidly to 30% after 1700 nm, and drops off again to below 20% after 2000 nm. A Model SLS202 extended wavelength tungsten-halogen light source from Thorlabs (Newton, New Jersey) with a peak output at 1500 nm, collimating optics, and a high extinction polarizer was used. Bandpass filters with varying wavelengths (bandwidths) of 1300 nm (90), 1460 nm (85), 1535 nm (80), and 1675 nm (90) were used. A polarized, broadband amplified spontaneous emission (ASE) light source Model AP-ASE-2000 from AdValue Photonics (Tucson, Arizona) with a center wavelength of 1959 nm, a bandwidth of ∼100  nm (−3  dB), 230 nm (−30  dB), and an output power of 11 mW was used for the 1950-nm light source. The light sources were placed at 20-deg angles to the camera, as shown in Fig. 2, but positioned on the same side. Images were processed and automatically analyzed using a dedicated program constructed with LabVIEW software.

### 2.4 SWIR Dehydration Analysis

Four parameters were used to characterize the dehydration curves that were acquired: delay, ΔI, OGR, and $\%I_{\text{fin}}$. There was an initial delay between the time the air was turned on and the rise in reflectivity; this delay was measured in seconds. The overall intensity change for the curve from 0 to 60 s was ΔI. The curves were fitted to a sigmoid function [Eq. (1)], where a–c are coefficients and I(t) is the intensity value at time (t) in seconds that represents the rate of growth of the curve or the change in reflectivity and dehydration. The Levenberg–Marquardt algorithm was used to estimate the best fit of each curve to the equation. The overall growth rate (OGR) of the function was given by a/b.^11^^,^^12^ Each curve was fit using Igor Pro from Wavemetrics (Portland, Oregon), and the OGR was calculated.

$$I(t)=\frac{a}{1+e^{\frac{c-t}{b}}}+d.$$ (1)

The fraction of the intensity change that takes place in the tail end of the curve after the initial rapid rise in reflectivity ($\%I_{\text{fin}}$) was also calculated using Eq. (2). The time of maximum change ($t_{\max}$) was calculated by taking the derivative of the dehydration curve and identifying the position of maximum slope. $\%I_{\text{fin}}$ is the change in intensity from time zero to $t_{\max}+10\;s$ divided by the change in intensity from time $t_{\max}+10$ to 60 s times one hundred.

$$\%I_{\text{fin}}=(\frac{I_{T\max+10}-I_{T0}}{I_{\text{End}}-I_{T\max+10}})*100.$$ (2)

Differences between the four parameters ΔI, delay, OGR, and $\%I_{\text{fin}}$ were compared using t-tests. Differences between the wavelengths were compared using one-way analysis of variance with repeated measures (RM-ANOVA) with Tukey’s multiple comparisons post-test. Linear and multivariate regression were used to compare ΔI, delay, OGR, and $\%I_{\text{fin}}$ with the lesion depth and surface zone thickness measured with microCT. A significant difference (p<0.05) from a line with a slope of zero indicated correlation (Pearson), and $R^{2}$ values indicate the strength of the correlation. Instat and Prism statistical software from GraphPad Software, Inc., (La Jolla, California) was used for the calculations. The significance level was set at p<0.05.

## 3 Results

**Fig. 3:**
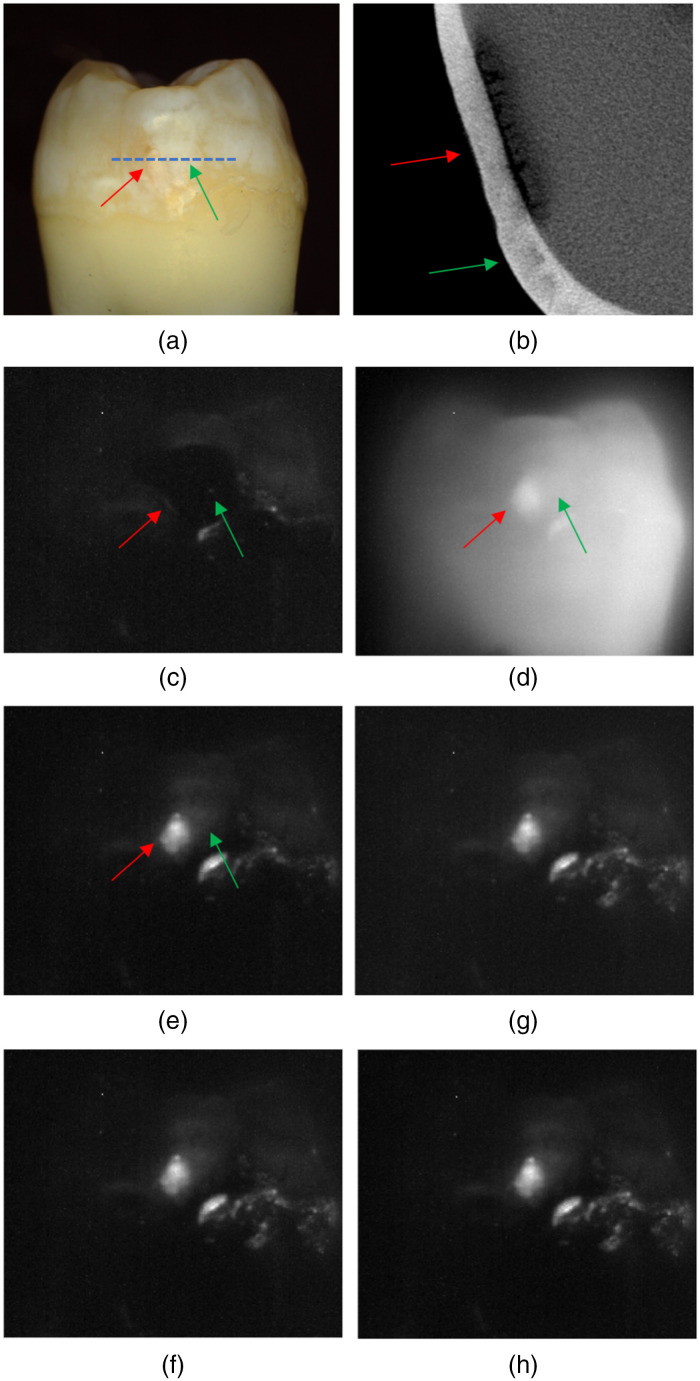
(a) Visible image of a tooth with active (red arrow) and arrested (green arrow) lesion areas. (b) MicroCT image of the area aligned with the blue dotted line in (a). SWIR images taken at (c) 1950 nm and (d) 1300 nm before dehydration. SWIR images at 1950 nm acquired after (e) 10; (f) 20; (g) 30; and (h) 60 s of dehydration.

**Fig. 4:**
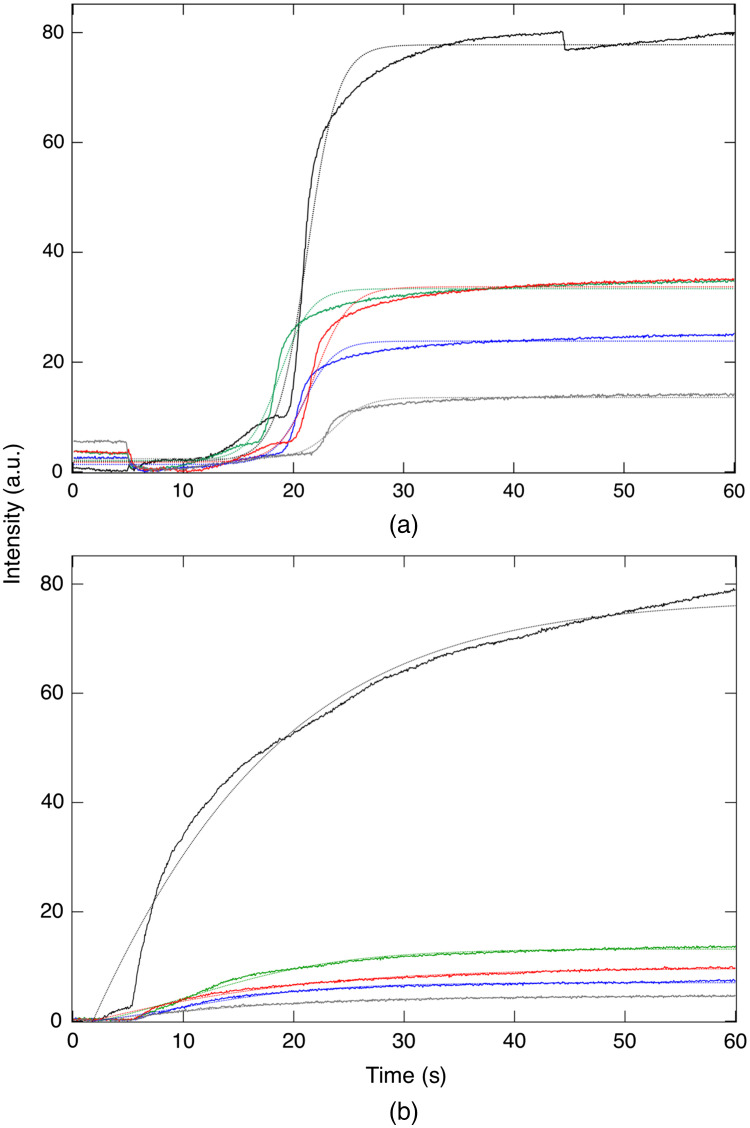
Dehydration curves at each wavelength for active (a) and arrested (b) lesion areas. The wavelengths listed from top to bottom are 1950—black, 1460—green, 1675—red, 1535—blue, and 1300—gray. The air was turned on at 0 s to initiate dehydration. Best fits of Eq. 1 are plotted for each curve.

**Fig. 5:**
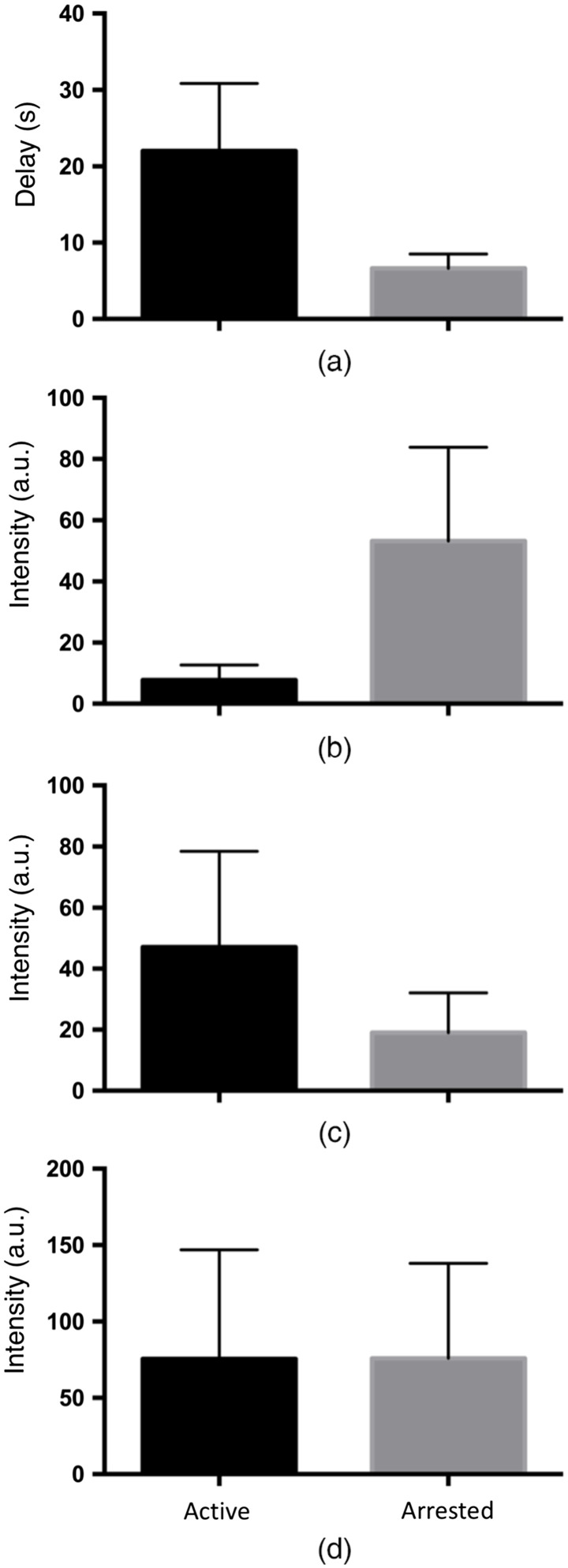
Plots of the mean±sd of (a) delay; (b) $\%I_{\text{fin}}$; (c) OGR; and (d) Δ*I* for the active (n=10) and arrested (n=10) lesion areas. The OGR, $\%I_{\text{fin}}$, and delay were significantly different (P<0.05) for active versus arrested lesions.

Time-sequence SWIR images of a lesion with both active and arrested lesion areas acquired during drying are shown in Fig. 3. Images of the tooth are shown at 1300 and 1950 nm before dehydration. At 1950 nm, the sound tooth surface is very dark with near-zero intensity, and the outline of the tooth is not visible. There is only some scattered light from suspected areas of demineralization and hypomineralization. At 1300 nm, the scattered light intensity from sound areas of the tooth is much higher, and the entire tooth surface can be seen. The red arrow in Fig. 3(c) points toward the area of an active lesion that is not visible initially in the initial 1950-nm image [Fig. 3(c)] but can be seen in the 1300-nm image [Fig. 3(d)]. The intensity of the active lesion (red arrow) increases rapidly during drying and within 10 s is the brightest area in Fig. 3(e). There are several areas on the tooth with increased scattering where the intensity increases after drying but the change is not as rapid. The green arrow points at an area that may be an arrested lesion or hypomineralization. It appears as a shallow band with a highly mineralized surface zone in the microCT image [Fig. 3(b)] in contrast to the active lesion area (red arrow) where the lesion penetrates into the dentin and no highly mineralized surface zone is visible. Images taken at later times (20 to 60 s) do not show as obvious changes as those observed in the first 10 s. The sound areas of the tooth are still not visible even after 60 s of drying. When dry, the contrast is markedly higher from the lesion areas at 1950 nm compared with other wavelengths, which is consistent with what has been observed in other recent studies.^20^^–^^22^ Dehydration curves for the SWIR wavelengths investigated in this study are plotted in Fig. 4 for an arrested lesion and an active lesion along with best-fit curves using Eq. (1). The intensity change, ΔI, is markedly higher for 1950 nm. The active lesion shows a distinct delay of 10 to 20 s before the change in reflectivity occurs, while the corresponding delay for the arrested lesion is on the order of 5 to 10 s. The change in intensity occurs more rapidly for the active lesion and reaches a plateau after 20 s. In contrast, the intensity change for the arrested lesion occurs much more gradually and does not plateau after 60 s. Based on this behavior, we employed four parameters to describe the dehydration curves: the delay before the reflectivity increases, the overall intensity change in a given time interval ΔI, the rapid rise or OGR of the curve, and the rise in reflectivity in the tail portion of the curve $\%I_{\text{fin}}$. The mean±sd of these four parameters is plotted in Fig. 5 for the active and arrested 1950-nm curves. There was no significant difference between active and arrested lesions for ΔI. However, there was a significant difference in ΔI for 1300 nm. A t-test showed that there was a significant difference between active and arrested lesions (p<0.05) for the delay, OGR, and $\%I_{\text{fin}}$. The largest differential was for $\%I_{\text{fin}}$. The mean±sd of these four parameters for all wavelengths along with the lesion contrast after 60 s of drying is tabulated in Table 1. Only 1950 nm had a significant difference for 3 out of the 4 parameters. The lesion contrast was significantly higher at 1950 nm (P<0.001) than for other wavelengths for both active and arrested lesions.

**Fig. 6:**
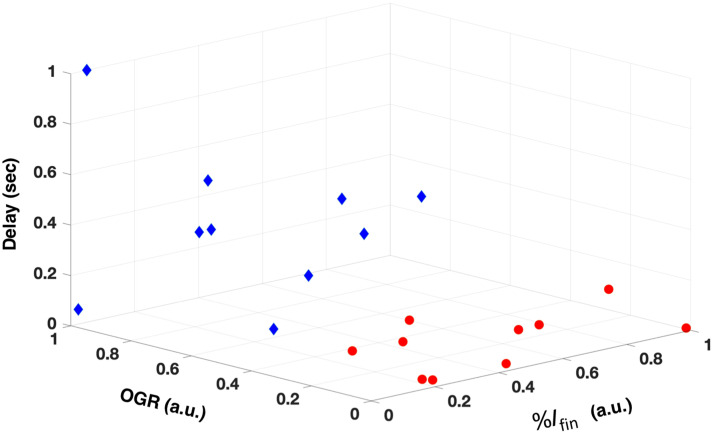
3D scatter plot of the $\%I_{\text{fin}}$, OGR, and delay. Red circles are arrested lesions, and blue diamonds are active lesions.

**Table 1:** Mean±sd of the delay, $\%I_{\text{fin}}$, OGR, Δ*I*, and lesion contrast at 60 s for the active (n=10) and arrested (n=10) lesion areas.

	1300 nm		1460 nm		1535 nm		1675 nm		1950 nm	
	Active	Arrested	Active	Arrested	Active	Arrested	Active	Arrested	Active	Arrested
Delay	35±11*	7.8±1.0	24±6.4*	6.3±0.2	23±6.2*	6.8±0.6	23±3.4*	10±3.8	22±2.8*	6.6±0.6
$\%I_{\text{fin}}$	5.6±8.6	22±9.3	30±24	28±6.3	67±61	25±5.2	11±4.5	39±28	7.6±1.6	53±10*
OGR	2.2±0.8	4.6±1.4	6.7±1.7	8.8±2.9	6.9±1.8	11±4.7	4.4±1.5	5.1±1.7	47±9.9*	19±4.1
Δ*I*	5.2±1.4	13±4.9*	15±3.9	25±8.2	13±3.4	25±9.0	9.7±2.6	15±5.1	76±23	75±19
Contrast	0.06±0.1	0.11±0.1	0.38±0.1	0.44±0.1	0.28±0.1	0.44±0.1	0.23±0.1	0.44±0.1	0.71±0.1	0.68±0.1

* An asterisk indicates that value is significantly higher (P<0.05).

The mean lesion depth was 538±284 for the ten active lesions and 680±424 for the ten arrested lesions. The mean surface zone thickness was 0 for the active lesions and 117±26.5 for the ten arrested lesions. There was no correlation of any of the four parameters with the lesion depth. There was correlation (P<0.05) of the $\%I_{\text{fin}}$, OGR, and delay parameters with the surface zone thickness with correlation coefficients ($R^{2}$) of 0.53, 0.29, and 0.58, respectively. Multivariate regression of the surface zone thickness and $\%I_{\text{fin}}$, OGR, and delay yielded an $R^{2}$ of 0.75 (P<0.0001). There was no correlation between $\%I_{\text{fin}}$, OGR, and delay. A 3D scatterplot of $\%I_{\text{fin}}$, OGR, and delay for the active lesions (blue) and arrested lesions (red) is shown in Fig. 6. There is clear separation in grouping between the active and arrested lesions in the 3D plot.

## 4 Discussion

The main objective of this study was to assess the performance of SWIR dehydration measurements at the strong water absorption band of 1950 nm on naturally formed lesions on extracted teeth. In prior studies filtered tungsten-halogen light sources did not have sufficient intensity at 1950 nm to perform these measurements, and scanned narrow-band lasers are poorly suited for the time-resolved measurements due to speckle noise. The high-power, polarized, broadband 1950-nm ASE source was ideally suited for these measurements. We observed very large changes in intensity (ΔI) after drying for 60 s at 1950 nm, and these changes were significantly higher than other wavelengths in the range from 1500 to 2060 nm, suggesting that the 1950-nm wavelength band is advantageous for dehydration measurements on tooth coronal surfaces to assess lesion activity. A significant concern regarding the high magnitude of the water absorption band at 1950 nm was that any residual water would greatly lower contrast and that it would be difficult to sufficiently dehydrate the lesions in a reasonable period of time. The time used for dehydration in this study was quite long 60 s; however, higher air pressures can also be investigated to accelerate dehydration. The air pressure for dehydration was set at 25 psi which is fairly low compared with the 50 to 80 psi air pressures typically set for the dental air-water syringe. In addition, the large distance of the nozzle for the *in-vitro* system (4 cm) used in this study yields a much lower effective pressure at the lesion surface, whereas the nozzle located on clinical probes is located less than a centimeter from the lesion during dehydration.^17^

In this study, there was no significant difference in ΔI between the active and arrested lesions at 1950 nm or for most of the other wavelengths, with only 1300 nm showing a significant difference. This result conflicts with previous studies, including the recent study on bovine enamel blocks^21^ where ΔI was significantly higher for active lesions. In this study, the lesions were chosen based on microCT data and are much deeper than the lesions that were chosen for previous investigation that utilized measurements with optical coherence tomography, which has a more limited imaging depth.^12^ Many of the lesions chosen in this study had depths greater than a mm, while the simulated lesions used on bovine and human enamel blocks have maximum depths of only 200  μm with the lesion depths between the active and arrested/remineralized samples having similar depths.^11^^,^^21^ Considering that the contrast of caries lesions depends on the lesion depth and severity, it is not surprising that, for a sample of 20 lesions with highly variable lesion depths, the overall reflectivity of the lesion would not be dominated by the presence or absence of a surface zone. Moreover, the mean depth of the arrested lesions was 21% higher, 0.68 versus 0.54 mm for the arrested and active lesions, respectively. The magnitude of ΔI also varies with the time of dehydration, and the difference between the active and arrested lesions decreases with longer dehydration times since the maximum reflectivity is more rapidly reached for active lesions due to the more rapid diffusion of water from the open pores. In addition, even though several studies have shown that mean ΔI values are significantly higher for active versus arrested lesions, the reflectivity after drying and ΔI are not practical measures for differentiating active from arrested lesions, and other measures such as the shape of the dehydration curve are better suited to differentiating active from arrested lesions. Arrested lesions appear to take longer to completely dry due to the lower permeability. Use of ΔI to discriminate between active and arrested lesions is not well suited for *in-vivo* measurements since the depth and severity of the lesions are not known ahead of time. The other three parameters, $\%I_{\text{fin}}$, OGR, and delay, are less dependent on the lesion depth and severity and better suited for clinical use.

We observed some notable differences in the evaporation dynamics from those observed in previous studies. The active lesions manifest a delay of several seconds before there is a change in intensity. This can be clearly seen upon comparing the 1950-nm curves in Figs. 3(a) and 3(b). Active lesions are typically rougher and more likely to have micro-cavitation than arrested lesions, and such surfaces are likely to have more water near the porous surface that takes a longer initial period to remove. OGR reflects the rate during the rapid rise in reflectivity as the lesion dehydrates; we used this approach previously^12^ and found that it was more effective than using ΔI to discriminate between active and arrested lesions. Arrested lesions take longer to completely dry compared with suspected active lesions, and the longer time to completely dehydrate the lesion was more noticeable for 1950 nm than for other wavelengths. The use of $\%I_{\text{fin}}$ worked well for quantifying the delay, and $\%I_{\text{fin}}$ gave better discrimination between active and arrested lesions at 1950 nm than the other parameters. $\%I_{\text{fin}}$ and OGR both depend on the shape of the dehydration curve, but they are independent parameters. $\%I_{\text{fin}}$ was even more effective for longer dehydration times. We recorded dehydration curves for as long as 2 to 4 min; however, longer dehydration times are not practical for clinical use.

## 5 Conclusions

In summary, this study has shown that dehydration measurements at 1950 nm are highly sensitivity to changes in water content and provide important insight into the evaporation dynamics in these samples. In addition, new approaches have been introduced to describe the dehydration process, namely the initial delay before the rapid rise in reflectivity and $\%I_{\text{fin}}$, which provide improved discrimination between active and arrested lesions. A multivariate approach including the three parameters $\%I_{\text{fin}}$, delay, and OGR provided the best correlation with the surface zone thickness that forms as the lesion is arrested.

## Code, Data, and Materials Availability

There is no code essential for reproducibility or supplementary data available for this manuscript.
